# Rare Presentation of Homozygous S*LC20A2* Mutations Causing Intra‐Arterial Cerebral Vasculopathy and Stroke in Infancy: Case Report and Review of the Literature

**DOI:** 10.1155/crig/1587968

**Published:** 2025-12-26

**Authors:** Laila Baker, Faisal Hadid, Sara Salahaldeen Irshaidat, Sondos Altaraqji, Ruba Benini, Farouq Thabet, Rana Al Shami, Husam Kayyali, Rehab Al Saleh, Tawfeg Ben Omran, Jehan Al Rayyahi, Ahmed El Beltagi, Baha Juma, Khalid Ibrahim

**Affiliations:** ^1^ Department of Pediatrics, Hamad Medical Corporation, P.O. Box 3050, Doha, Qatar, hamad.qa; ^2^ Department of Pediatrics, Sidra Medicine, P.O. Box 26999, Doha, Qatar, sidra.org

**Keywords:** arterial vasculopathy, Fahr′s disease, primary familial basal ganglia calcification, *SLC20A2* pathogenic variant homozygous

## Abstract

Primary familial basal ganglia calcification (PFBC), also known as Fahr’s disease, is a rare neurodegenerative condition characterized by bilateral calcifications in the basal ganglia and other brain regions. While it predominantly presents in adulthood and is commonly associated with heterozygous mutations, rare homozygous pathogenic variants may lead to severe early‐onset manifestations. We present a unique case of PFBC in a 2‐month‐old female infant who exhibited focal motor seizures shortly after routine immunization. Neuroimaging revealed bilateral basal ganglia calcifications, multifocal cerebral infarcts, and evidence of significant intra‐arterial cerebral vasculopathy. Genetic testing confirmed a homozygous pathogenic variant in the *SLC20A2* gene (*c.1399 C > T*) (*p.R467*∗). Notably, both consanguineous parents were heterozygous carriers of the same mutation. Other family members across two generations also harbored heterozygous variants but were asymptomatic. This case is one of the few reported instances of a homozygous *SLC20A2* pathogenic variant and the second to demonstrate intra‐arterial vasculopathy in infancy. The clinical spectrum included seizures, hypotonia, poor feeding, and extensive ischemic changes. Despite supportive care and antiepileptic therapy, the patient remained neurologically impaired. This report underscores the importance of considering PFBC in the differential diagnosis of infantile seizures, especially in populations with high rates of consanguinity. It also expands the phenotypic spectrum of *SLC20A2*‐related disease to include infantile stroke due to cerebral vasculopathy. Early diagnosis and genetic counseling are essential for management and family planning.

## 1. Introduction

Primary familial basal ganglia calcification (PFBC), also known as striopallidodentate calcinosis, was initially identified by German neurologist Karl Theodor Fahr in 1930. However, the first documented case of bilateral basal ganglia calcification dates back to 1850, described by Delacour in a 56‐year‐old man exhibiting stiffness, weakness of lower extremities, and tremors [1, 2].

PFBC is characterized by idiopathic calcification and the deposition of zinc, aluminum, and magnesium in a symmetric pattern within the basal ganglia. This calcification may extend to other brain regions, including the cerebellar dentate nuclei, thalami, subcortical white matter, cerebral, and cerebellar cortex [3].

The prevalence of PFBC is estimated to range from 2.1 to 6.6 per 1000 persons [4, 5]. Pathophysiologically, it is believed to stem from abnormalities in calcium–phosphorus metabolism, functional and microanatomical alterations of pericytes, dysfunction of the blood–brain barrier (BBB), and the creation of an osteogenic environment leading to astrocyte activation and progressive neurodegeneration [6].

While PFBC typically presents between the ages of 40 and 60, late‐onset cases have been documented, such as a 77‐year‐old man presenting with increasing confusion, altered mental status, dystonia, tremors, and hallucinations [7].

The clinical spectrum of PFBC is broad, ranging from asymptomatic to neuropsychiatric and movement disorders. Symptoms may include seizures, headaches, gait abnormalities, cerebellar dysfunction (such as vertigo and dystonia), sensory abnormalities, early dementia, confusion, mood disturbances, personality changes, catatonia, cognitive impairment, psychosis, irritability, and aggression. Approximately one‐third of pathogenic variant carriers may remain clinically unaffected. Notably, parkinsonism and speech disturbances are frequently reported motor manifestations, while cognitive deficits, headaches, and depression are major nonmotor symptoms/signs [8].

The criteria for diagnosing Fahr’s disease typically include (1) evidence of bilateral basal ganglia calcification through computed tomography (CT) or magnetic resonance imaging (MRI), which reveals calcifications in the basal ganglia, usually symmetrically distributed, and (2) progressive neurological or neuropsychiatric manifestations [9–11].

Meeting both criteria, particularly in the absence of other underlying causes for basal ganglia calcification, can support a diagnosis of Fahr’s disease. It is important to exclude secondary causes of basal ganglia calcification, such as metabolic disorders, infections, or trauma, which will cause Fahr syndrome (Table 1).

**Table 1:** Secondary causes of basal ganglia calcification.

Secondary causes to be excluded:
1. Infectious causes including TORCH infection.
2. Endocrine causes: hypothyroidism–hypoparathyroidism.
3. Poisoning with toxic compounds (CO, methanol, cyanide)
4. Liver disorders (hepatic encephalopathy or Wilson’s disease)
5. Vascular pathologies (hypertensive infarcts or embolic events)
6. Malignant neoplasms (primary central nervous system lymphoma)
7. Metabolic/mitochondrial disorders.

PFBC cases can either occur sporadically or be inherited with an autosomal dominant (AD) pattern with incomplete penetrance or an autosomal recessive (AR) pattern. Dominantly inherited PFBC is linked to pathogenic variants in four genes: solute carrier 20 member 2 (*SLC20A2*), xenotropic and polytropic retrovirus receptor 1 (XPR1), platelet‐derived growth factor B (PDGFB), and platelet‐derived growth factor receptor B (PDGFRB). Recessively inherited PFBC is caused by pathogenic variants in three genes: Myogenesis Regulating Glycosidase protein (*MYORG*), Junctional Adhesion Molecule 2 (*JAM2*), and cytidine monophosphate (*UMP-CMP*) kinase 2 (*CMPK2*) [12–19].

In this context, we present a case of a child with intra‐arterial vasculopathy and a homozygous pathogenic variant in the *SLC20A2*, demonstrating an early and severe phenotype (see Table 2). The first report was by Arteche for a 55‐year‐old female with a history of vertigo in her twenties and dysarthria in her forties, presenting with motor symptoms such as clumsiness, balance disturbance, and asymmetric parkinsonism, accompanied by psychosis. MRI brain imaging revealed extensive cortical bihemispheric calcifications in the basal ganglia, periventricular white matter, posterior cortex, and cerebellum [20].

**Table 2:** Clinical spectrum of recessive *SLC20A2*‐related PFBC.

	Arteche‐Lopez 2021 [20]	Ceylan 2022 [21]	Ceylan 2022 [21]	D’Onofrio 2023 [22]	Our patient 2024
Gender	F	F	M	M	F
Consanguinity	Yes	Yes	Yes	Yes	Yes
Carrier parents	Healthy (brain CT not performed)	Healthy mother, father with migraine (MRI for both parents revealed symmetric signal loss on the SWI sequence at the bilateral basal ganglia	Healthy mother, father with migraine (MRI for both parents revealed symmetric signal loss on SWI sequence at the bilateral basal ganglia	Healthy (brain CT not performed)	Affected asymptomatic (brain CT not performed)
Variant	*c.211c > T*	*c.1794+1G > A*	*c.1794+1G > A*	*c.560A > g*	Homozygous pathogenic variant in the *SCL0A2* gene (*c.1399 C > T*)
Protein	*p.(Arg71Cys)*	*p.Ser570Argfs∗30*	*p.Ser570Argfs∗30*	*p.(Tyr187Cys)*	*p.R467*∗
Exon/intron	Exon 2	Intron 10	Intron 10	Exon 5	
Symptom’s onset	20 y	Birth	Birth	2 m	2 m
Age at follow‐up	55 y	2 y 5 m	13 m	5 y	3 m
Psychomotor delay	No	Yes	Yes	Yes	Yes
Epilepsy	No	Yes	Yes	Yes	Yes
Language	Dysarthria > 40 y	NR	NR	Nonverbal communication	NA
Motor involvement	Clumsiness, balance disturbance, asymmetric parkinsonism 40–50 y	Spasticity in four limbs	Hemiplegic right side	Spastic tetraparesis (right > left)	Hypotonia
Psychiatric symptoms	Psychosis 8 y (paranoid delirium improved on aripiprazole)	NR	NR	No	No
Neuroimaging findings	Extensive cortical bihemispheric calcification in BG, periventricular WM, posterior cortex and cerebellum	Cerebral atrophy, abnormal signal changes in cerebral, possible microcalcifications and subdural effusion	Chronic sequel changes in both cerebral hemispheres, especially in the posterior parts and ventricular dilatation	Severe anterior and posterior moyamoya vasculopathy, T1 hyperintensity of the globi pallidi and BG, cortical–subcortical and WM calcification	Infarct involving the right parieto‐occipital region. Small acute ischemic foci of the frontal subcortical WM. Intracranial calcified arterial vasculopathy involving the circle of Willis and major cerebral arteries. Evident significant stenosis seen on the MRA. Foci of parenchymal and extra‐axial mineralization.
Dysmorphism	NR	NR	NR	Minor (small‐spaced teeth, thickened tragus and lobes, synophrys)	None
Other features	NR	Bilateral congenital cataracts, microcephaly, growth retardation	Bilateral congenital cataracts, microcephaly, growth retardation, secundum atrial septal defect	Bilateral congenital cataracts, acquired microcephaly	Swallowing difficulties

*Note:* F, female; M, male; m, month; y, year.

Abbreviations: BG, basal ganglia; CT, computed tomography; MRA, magnetic resonance angiography; NR, not reported; SWI, susceptibility‐weighted imaging; WM, white matter.

Subsequently, Ceylan reported a case involving a biallelic *SLC20A2* variant with a splicing effect in two siblings aged 2 years and 5 months and 13 months, respectively. Both siblings presented with symptoms resembling cytomegalovirus (CMV) infection, including brain calcification, growth retardation, bilateral cataracts, microcephaly, and convulsions. Interestingly, the parents were healthy, although the father exhibited migraine symptoms. MRI head scans of both parents revealed symmetric signal loss on the SWI sequence at the bilateral basal ganglia [21].

Another report by D’Onofrio described a case of a 2‐month‐old boy born to consanguineous healthy parents. The boy initially presented with focal motor seizures, which progressed to spastic tetraparesis, acquired microcephaly, and severe psychomotor delay [22].

Currently, symptomatic management remains the only available option, and calcium‐chelating agents are still under research.

## 2. Case Report

We present a case of a 2‐month‐old female infant, born at term by cesarean section (CS) due to a previous scar. The mother had gestational diabetes, but otherwise, she had an unremarkable prenatal, perinatal, and natal history.

One day after she received her 2‐month vaccines, she presented to the pediatric emergency center (PEC) with prolonged episodes of abnormal movements with eyes flickering and jerky movements of the left upper and lower limbs associated with bluish discoloration of the face.

She was managed as per the convulsive status epilepticus protocol, receiving lorazepam followed by a loading dose of levetiracetam; however, as she continued to seize, she was admitted to the pediatric intensive care unit (PICU), where loading doses of phenobarbitone and phenytoin were given, and then, she was commenced on maintenance doses of levetiracetam and phenobarbitone.

Initial blood workup including complete blood count (CBC), comprehensive metabolic panel (CMP), and CRP was unremarkable. Ultrasound head was remarkable for bilateral symmetrical linear densities noted in the basal ganglia, most likely representing mineralizing vasculopathy.

EEG showed mild cerebral disturbance in the left parietotemporal area. Initial brain MRI/MRA/MRV showed multifocal cerebral, recent infarcts more at the right parieto‐occipitotemporal area, likely hemodynamic multiterritory hypoxic ischemic changes, possibly related to proximal intracranial vasculopathy/arteriopathy with significant attenuation of the circle of Willis, and proximal anterior and posterior circulation main branches.

At this point, our genetics team was involved, and further history and data were obtained, which revealed that a maternal aunt was diagnosed with Fahr’s disease when she was in her 40s.

**Figure 1:**
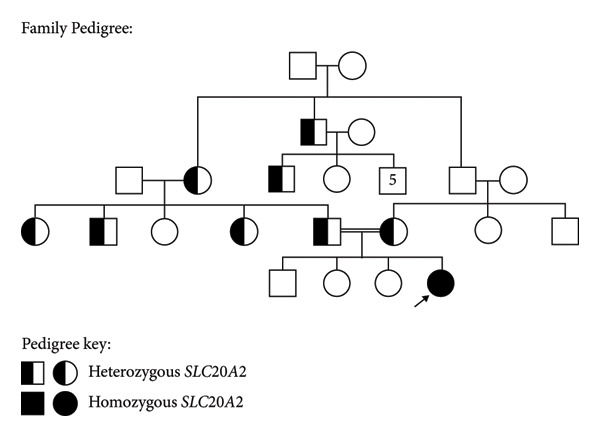
Family pedigree. Whole exome sequencing: homozygous pathogenic variants in the *SLC20A2* gene (*c.1399 C >* *T*) (*p.R467*∗).

Both parents, who are first‐degree cousins, are heterozygous for a pathogenic variant in the *SLC20A2* gene, associated with Fahr’s disease. The three siblings of the child are healthy (Figure 1).

Repeated brain MRI/MRA and CT head showed “an expected” interval evolution of the previously noted bilateral ischemic changes, including the previously described dominant infarct involving the right parieto‐occipital region.

New small acute ischemic foci involving the frontal subcortical white matter were noted.

**Figure 2:**
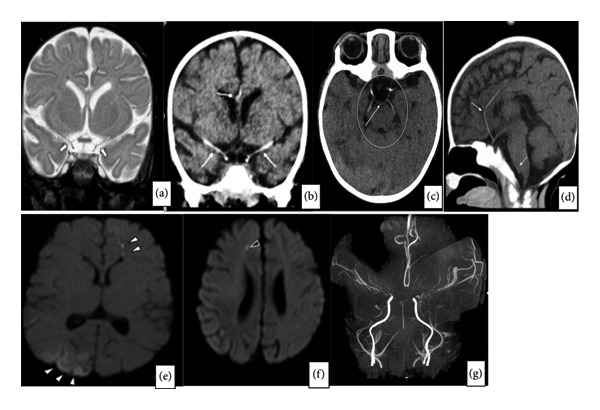
(a–g) Computed tomography (CT) of the head and magnetic resonance imaging (MRI) of the brain in our patient.

Significant intracranial calcified arterial vasculopathy involving the circle of Willis and major cerebral arteries is noted, with evidence of significant stenosis seen on the MRA. Several foci of parenchymal and extra‐axial mineralization were also noted. The extracranial neck vasculature appears unremarkable (Figures 2(a), 2(b), 2(c), 2(d), 2(e), 2(f), and 2(g)).

Coronal (Figure 2(a)), axial (Figure 2(b)), and sagittal (Figure 2(c)) images from an unenhanced CT study show significant calcification of the intracranial internal carotid arteries, circle of Willis, anterior cerebral arteries, middle cerebral arteries, and segments of the vertebrobasilar system (thin white arrows and circle). Coronal T2‐weighted image (Figure 2(d)) of the initial MR study shows markedly narrowed distal internal carotid arteries and proximal anterior and middle cerebral arteries (thick white arrows). The axial diffusion‐weighted image (Figure 2(e)) demonstrates diffusion restriction, denoting infarcts, involving the right parieto‐occipital cortical/subcortical region with a couple of smaller foci of infarcts within the left frontal deep white matter (white arrowheads). Follow‐up MRI 10 days later (Figure 2(f)) shows a new focus of diffusion restriction (infarct) within the right deep white matter (black arrowheads). Time‐of‐flight MR angiography (Figure 2(g)) demonstrates marked stenosis of the distal internal carotid arteries at their bifurcations as well as the A1 and M1 segments of the anterior and middle cerebral arteries, respectively. Localized stenosis at the confluence of the vertebral artery into the basilar artery was also noted as well as significant attenuation of the proximal posterior cerebral arteries.

The patient’s blood workup for coagulopathy was unremarkable. While there was evidence of elevated parathyroid hormone (PTH) and low vitamin D levels, the remainder of the endocrinological evaluation was otherwise normal.

Oral aspirin was started. In consultation with the hematology team, alternative anticoagulant therapies were not considered due to the potential risk of bleeding.

An echocardiogram was performed, which yielded unremarkable results. Additional imaging studies, including ultrasound and Doppler evaluations of the neck and abdomen, were also unremarkable, which helped rule out other potential causes of vasculopathy.

The patient remained clinically stable, with no further seizures. She exhibited normal limb movement without restriction. However, she was generally lethargic and showed signs of generalized hypotonia. Due to weak sucking and swallowing reflexes, she was fed via nasogastric tube.

The endocrinology team was consulted regarding the potential role of bisphosphonates in her management. After careful consideration, it was concluded that while bisphosphonates may carry potential benefits, they also pose risks of side effects. Therefore, their use in this case would be considered primarily for compassionate and palliative purposes, with uncertain outcomes.

Furthermore, the potential role of external revascularization procedures was thoroughly discussed and evaluated by our neurosurgical colleagues.

## 3. Discussion

To our knowledge, this is the first case report to describe homozygous pathogenic variants in *SLC20A2* presenting with pediatric stroke in the context of significant cerebral artery vasculopathy.

The report of Arteche was for an adult patient with different presenting symptoms from those seen in children. There was some predominance of movement disorder symptomatology for a 55‐year‐old female with a history of vertigo in her twenties and dysarthria in her forties, presenting with motor symptoms such as clumsiness, balance disturbance, and asymmetric parkinsonism, accompanied by psychosis. MRI brain also revealed extensive calcification but with a different distribution [20].

Subsequently, Ceylan reported a case involving a biallelic *SLC20A2* variant with a splicing effect in two siblings aged 2 years and 5 months and 13 months, respectively. Both siblings presented with symptoms resembling CMV infection, including brain calcification, growth retardation, bilateral cataracts, microcephaly, and convulsions. In those cases, the parents were healthy, although the father exhibited migraine symptoms. MRI head scans of both parents revealed symmetric signal loss on the SWI sequence at the bilateral basal ganglia, which was not the case in our patient’s parents [21].

The case reported by D’Onofrio and colleagues carries some similarity with ours with regard to the age at presentation and the seizure focality, but the subsequent course seems to be different. The child in their case became more spastic and more developmentally delayed [22] (Table 2).

Idiopathic basal ganglia calcification is a rare neurological disorder with variable presentation; however, it has been reported as a cause of ischemic stroke in two cases.

In the first case, the 36‐year‐old male presented with left hemiparesis, mild dysarthria, and left central facial palsy. Imaging revealed extensive and symmetric calcifications involving various brain regions, including the basal ganglia, internal capsules, thalami, cerebral subcortical white matter, cerebellar dentate nuclei, and deep cerebellar white matter. Additionally, MRI showed an acute ischemic infarction in the right posterior limb of the internal capsule. Management included aspirin therapy [23].

The second case involved a 66‐year‐old patient who presented with right conjugate deviation, right hemiparesis, and total aphasia following a convulsive seizure. Brain imaging also revealed symmetric calcifications in the basal ganglia, thalamus, and cerebellar dentate nuclei consistent with IBGC. Diffusion‐weighted MRI showed multiple small infarctions in the bilateral cerebral subcortical area [24].

The presentation of idiopathic basal ganglia calcification, also known as PFBC, can indeed vary widely, ranging from asymptomatic cases to severe manifestations. It is notable that approximately 50% of PFBC patients do not exhibit pathogenic variants in the seven currently known genes associated with the condition [25].

An intriguing aspect is that due to advancements in neuroimaging and sequencing technologies, around one‐third of patients who harbor pathogenic variants in PFBC genes may remain clinically asymptomatic throughout their lives. This suggests that factors beyond genetic mutations play a role in determining the clinical expression of the condition.

The penetrance, or the proportion of individuals carrying a specific genetic variant who exhibit signs and symptoms of the associated condition, varies among different PFBC genes. For instance, genes like *PDGFB*, *MYORG*, and *JAM2* exhibit high clinical penetrance, with more than 85% of carriers showing symptoms. In contrast, *PDGFRB* has the lowest clinical penetrance at 46%. XPR1 and *SLC20A2* have penetrance of 70% and 60%, respectively [26].

The observed clinical variability and reduced penetrance could be influenced by other genetic modifiers that have yet to be identified. This complexity underscores the need for further research to fully understand the underlying mechanisms driving PFBC and its diverse clinical presentations.

While MRI provides superior anatomical information, noncontrast CT is currently considered the best imaging modality for detecting brain calcifications.

The pattern of brain calcification in PFBC can indeed vary among different genes associated with the condition. Patients with biallelic variants in the *MYORG* and *JAM2* genes tend to exhibit more extensive areas of calcification compared to those with AD PFBC genes.

Distinctive features have been observed in PFBC related to *MYORG*. These include calcification in the brainstem, particularly in the pons, along with varying degrees of cerebellar atrophy. On the other hand, patients with *JAM2* mutations often present with more severe and confluent bilateral parietal, temporal, and occipital cortical calcifications.

It is interesting to note that more restricted brain calcification, primarily limited to the basal ganglia, can be found in individuals who carry monoallelic variants of *MYORG*, *JAM2*, and *CMPK2* genes. This suggests that the neuroradiological phenotype may be transmitted as a semidominant trait via heterozygous variants in AR PFBC genes [26].

## 4. Conclusion

The identification of intra‐arterial vasculopathy in conjunction with a homozygous *SLC20A2* mutation adds to the growing body of evidence linking genetic factors to vascular abnormalities in PFBC. Furthermore, our findings emphasize the need for increased awareness of PFBC among healthcare providers, especially in regions where consanguineous marriages are common. Early recognition of PFBC can facilitate timely interventions and improve patient outcomes by preventing complications associated with progressive vasculopathy.

## Consent

The patient’s parents allowed personal data processing, and informed consent was obtained from them.

## Conflicts of Interest

The authors declare no conflicts of interest.

## Author Contributions

Laila Baker and Faisal Hadid contributed equally to the work.

## Funding

Open access funding was provided by the Qatar National Library. Sidra Medicine Open Access publishing facilitated by the Qatar National Library, as part of the Wiley Qatar National Library agreement.
